# Correction to: MEMS-actuated metasurface Alvarez lens

**DOI:** 10.1038/s41378-020-00233-y

**Published:** 2021-02-01

**Authors:** Zheyi Han, Shane Colburn, Arka Majumdar, Karl F. Böhringer

**Affiliations:** 1 Department of Electrical and Computer Engineering, University of Washington, Seattle, WA 98195 USA; 2 Institute for Nano-Engineered Systems, University of Washington, Seattle, WA 98195 USA; 3 Department of Physics, University of Washington, Seattle, WA 98195 USA; 4 Department of Bioengineering, University of Washington, Seattle, WA 98195 USA

Correction to: *Microsystems & Nanoengineering*

10.1038/s41378-020-00190-6 published online 5 October 2020

The authors would like to add the below ‘Conflict of interest’ statement to this article^1^.

## Conflict of interest

“Arka Majumdar and Karl F. Böhringer are co-founders of Tunoptix Inc., which is commercializing technology discussed in this article.”

The original article has been updated.

## References

[1] Han Z (2020). MEMS-actuated metasurface Alvarez lens. Microsyst. Nanoeng..

