# Supplementary material for: MEMS-actuated metasurface Alvarez lens
Source: Microsyst Nanoeng. 2020 Oct 5;6:79. doi: 10.1038/s41378-020-00190-6 (PMC8433358; doi:10.1038/s41378-020-00190-6)
Supplement: Supplementary file 1 — Supplementary Information [file 41378_2020_190_MOESM1_ESM.docx]

# Supplementary Information: MEMS-actuated Metasurface Alvarez Lens

*Zheyi Han*^1^, email: [zh25@uw.edu](mailto:zh25@uw.edu)

*Shane Colburn*^1^, email: [scolbur2@uw.edu](mailto:scolbur2@uw.edu)

*Arka Majumdar*^1,2^, email: [arka@uw.edu](mailto:arka@uw.edu)

*Karl F. Böhringer*^1,3,4,*^, email: [karlb@uw.edu](mailto:karlb@uw.edu), phone: 206-221-5177, fax: 206-543-3842

^1^Department of Electrical and Computer Engineering, University of Washington, Seattle, Washington 98195, USA.

^2^Department of Physics, University of Washington, Seattle, Washington 98195, USA.

^3^Department of Bioengineering, University of Washington, Seattle, Washington 98195, USA.

^4^Institute for Nano-engineered Systems, University of Washington, Washington 98195, USA.

^*^Correspondence to: Karl F. Böhringer

## Metasurface Design


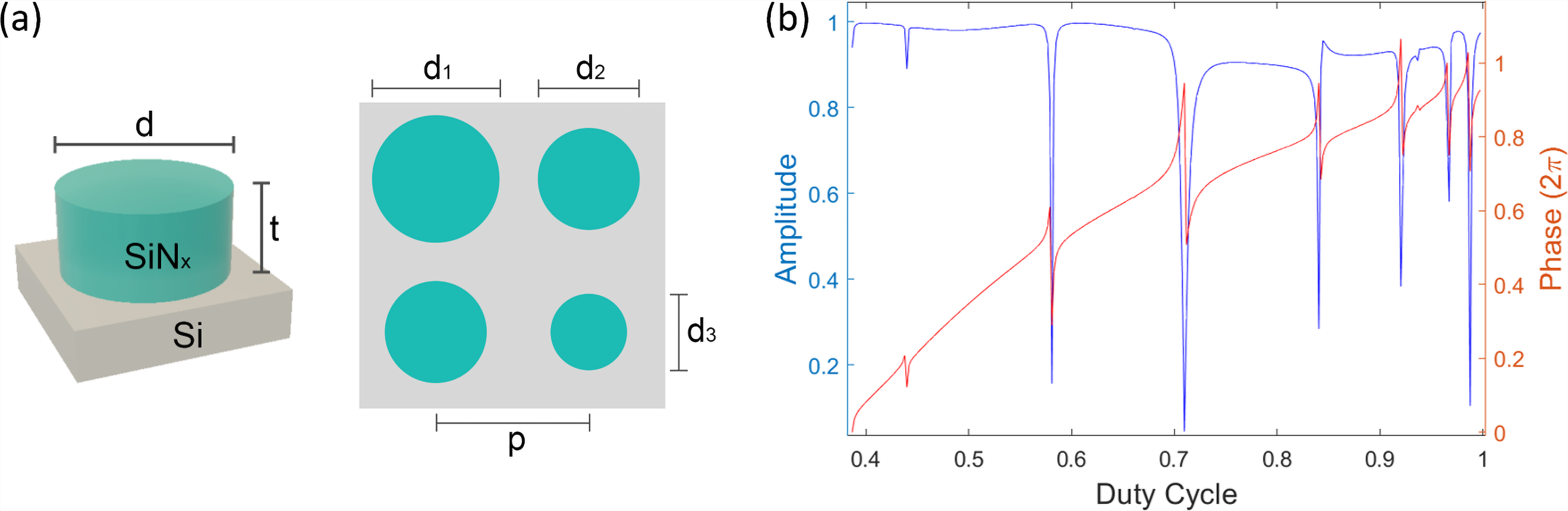


*Figure* ***S1.*** *(a) Schematics of a silicon nitride cylindrical nanopost with diameter d and thickness t on a silicon substrate, and top view of an exemplary metasurface array of a fixed periodicity p and varying nanopost diameters. (b) Simulated transmission phase and amplitude profiles of silicon nitride nanoposts as functions of duty cycle (d/p) for a chosen post thickness of 2 µm and a fixed array periodicity of 1.3 µm. The phase is normalized to 2π.*

## Device Fabrication


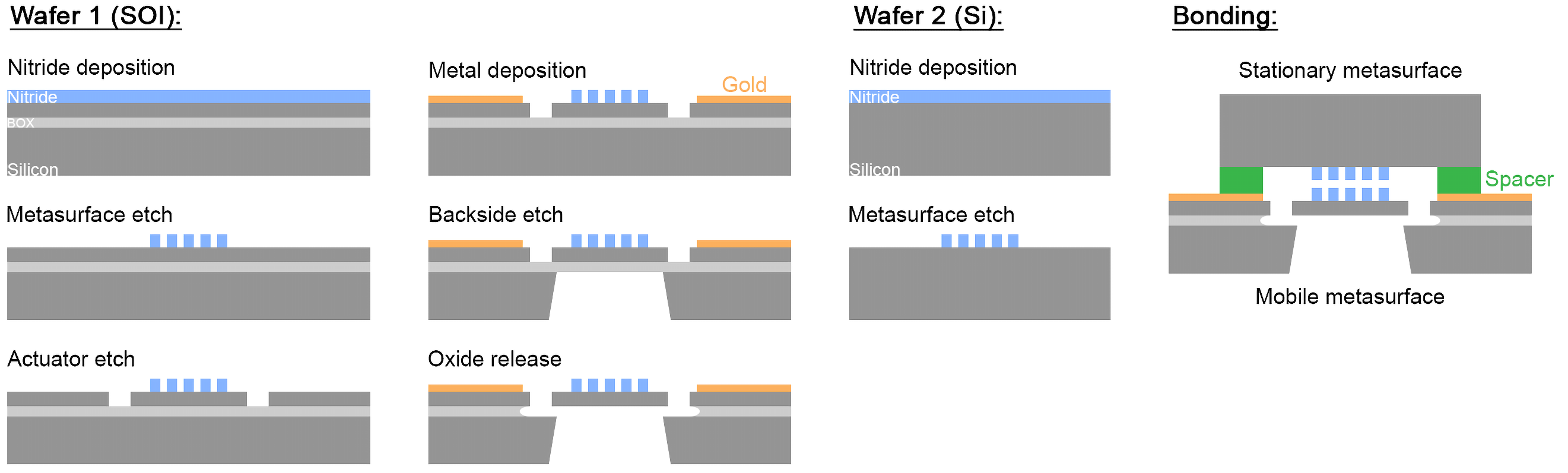


*Figure* ***S2.*** *Fabrication flow of a MEMS-actuated metasurface Alvarez lens.*

## Experimental Setup


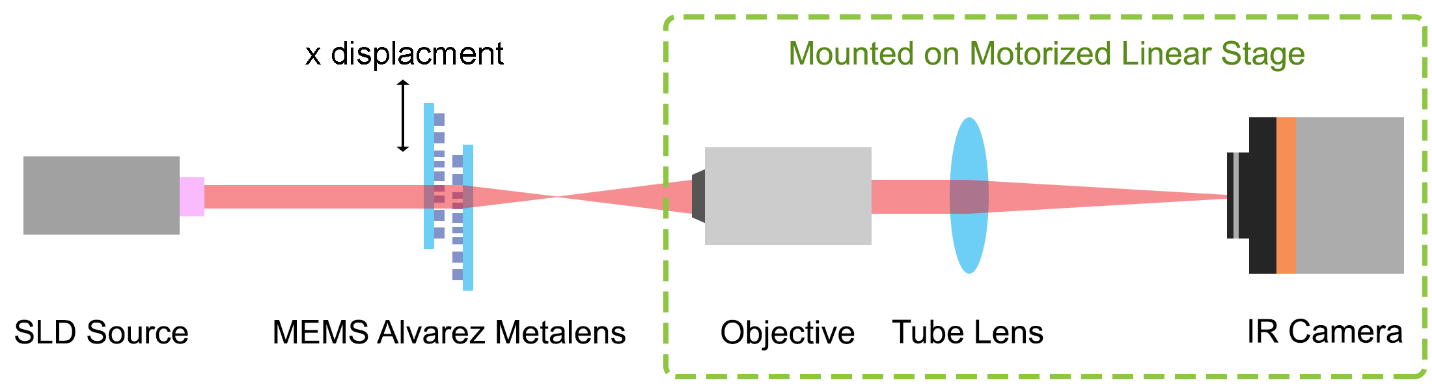


*Figure* ***S3.*** *Experimental setup for focal tuning analysis.*

## Raw Image Noise Reduction


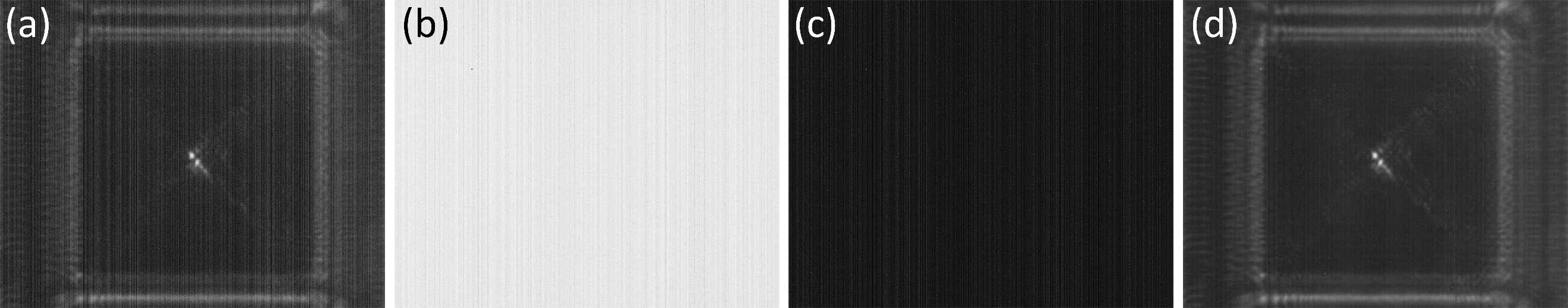


*Figure* ***S4.*** *Noise due to camera artifacts removed by reference subtraction. (a) A raw image captured by the IR camera. (b) A bright reference taken with a free light path. (c) A dark reference taken with the light path blocked. (d) The processed image with noise due to camera artifacts removed.*

Due to the intrinsic non-uniformity of the IR camera, the captured raw images contained vertical stripes as shown in Figure S4a. To remove the stripe patterns in the images, a bright reference (Figure S4b) was taken when the incident light directly illuminated the camera detector and a dark reference (Figure S4c) was taken when the incident light from the SLD source was completely blocked. The existence of static vertical stripes in the light and dark references identify them as systematic noise.

With the pixel intensities expressed in the double numeric data type (0 = black and 1 = white), the systematic noise subtraction is conducted pixel-wise to produce Figure S4d following the algorithm:

$$I_{processed}=I_{raw}+\left( {1-I}_{bright} \right){- I}_{dark}$$

This process also removed most image defects due to bad camera pixels.

## Experimental Data Error Analysis

The actuated displacement for the metasurface singlet lens was calculated as the averaged focal location shift at each applied voltage. Based on the frame rate of the source video for focal tracking and the defined linear ramping of applied voltage, the focal shift was analyzed from 33 frames for each voltage step. The error bar of each averaged displacement was calculated from the standard deviation of the 33 focal shifts within the same voltage step.

The actuated displacement at each applied voltage for the metasurface Alvarez lens was calculated as the average of all net platform displacements from valid edge detection at various locations in the corresponding screenshot. Its associated error was calculated from the standard deviation of displacements detected at multiple edges at each applied voltage.

The focal position along the optical axis at each applied voltage was found as the location of the weighted centroid of the high-intensity pixel regions above the defined thresholds, with the normalized intensity values as the weights. The corresponding associated uncertainty $\sigma$ was calculated using error propagation as the reciprocal square root of the sum of all the individual weights (normalized intensities) $w_{i}$ involved, mathematically expressed as^1^

$$\sigma=\frac{1}{\sqrt{\sum w_{i}}}$$

With the chosen intensity threshold percentiles of 0.1%, 0.3%, and 0.5%, the calculated uncertainties are all below 0.5 µm, hence it may be difficult to notice in the focal tuning plots in the main text. On one hand, a larger intensity threshold percentile value defines a more dispersed focal spot, which includes more pixels in the weighted centroid calculation. As described in the above equation, the increase in the size of the information pool for calculation leads to an increased sum of weights, and hence a smaller uncertainty. However, on the other hand, the definition of focal spot confines its footprint so the area of high-intensity pixels included for the calculation cannot be increased indefinitely. In applications, the area of the focal spot may need to be carefully defined case by case according to the operation requirements.

## Tilting of the Focal Axis with Asymmetric Actuation

A typical Alvarez lens should have the two cubic phase elements laterally actuated by the same distance simultaneously in the opposite directions. As described in the main text, to increase the robustness of our prototype Alvarez metalenses, only one of the complementary metasurfaces was fabricated on the MEMS-actuated platform to introduce the relative displacement between the pair. Such asymmetric actuation introduced a longitudinal shift of the focal plane along the optical axis and a lateral shift of the focal spot along the actuation direction^2^. To demonstrate this effect mathematically, we can derive the system’s phase function with one of the cubic phase plates being laterally actuated by a displacement $d$ along the $x$-direction while the other stays stationary. $d_{0}$ denotes the initial center offset we introduced to each metasurface in opposite directions to aid experimentation. Using the cubic phase expressions in Eq. (5) of the main text, the system’s phase function can be shown as

$$\varphi_{Alvarez}\left( x,y \right)=\varphi_{reg}\left( x+d_{0}+d,y \right)+\varphi_{inv}\left( x-d_{0},y \right)$$

$$=A\left[ \frac{1}{3}\left( x+d_{0}+d \right)^{3}+\left( x+d_{0}+d \right)y^{2} \right]-A\left[ \frac{1}{3}\left( x-d_{0} \right)^{3}+\left( x-d_{0} \right)y^{2} \right]$$

$$=A\left( 2d_{0}+d \right)\left( x^{2}+y^{2} \right)+Ad\left( 2d_{0}+d \right)x+A\left( \frac{2}{3}{d_{0}}^{3}+{d_{0}}^{2}d+d_{0}d^{2}+\frac{1}{3}d^{3} \right)$$

where the constant $A$ denotes the cubic phase strength.

Neglecting the constant phase terms, we can rewrite the system’s phase function as

$$\varphi\left( x,y \right)=A\left( 2d_{0}+d \right)\left( x^{2}+y^{2} \right)+Ad\left( 2d_{0}+d \right)x$$

We now interpret the phase function as a superposition of two different components, with the first term equivalent to a quadratic lens with a focal length at an operating wavelength $\lambda$ given by

$$f\left( d \right)=\frac{\pi}{\lambda A\left( 2d_{0}+d \right)}$$

The second term of the system’s phase represents a linear phase ramp in $x$ for a beam deflector with a deflection angle given by the generalized law of refraction^3^

$$n_{t}\sin\theta_{t}-n_{i}\sin\theta_{i}=\frac{\lambda_{0}}{2\pi}\frac{d\Phi}{dx}$$

Where $n_{i}$ and $n_{t}$ are the local indices of refraction, $\theta_{i}$ and $\theta_{t}$ are the angles of incidence and transmission, $\lambda_{0}$ is the vacuum wavelength and $\Phi$ is the abrupt phase shift at the interface.

Assuming operation in vacuum ($n_{i}=n_{t}=1$) and normal incidence ($\theta_{i}=0$), we can substitute the second term of our phase function for the abrupt phase shift, i.e., $\Phi=Ad\left( 2d_{0}+d \right)x$, and solve for $\theta_{t}$

$$\theta_{t}=\sin^{-1} \left[ \frac{\lambda_{0}Ad\left( 2d_{0}+d \right)}{2\pi} \right]$$

which represents the deflection angle of the focal axis.

## Alvarez Metalens with Manual Tuning to Explore the Effect of Aperture Size


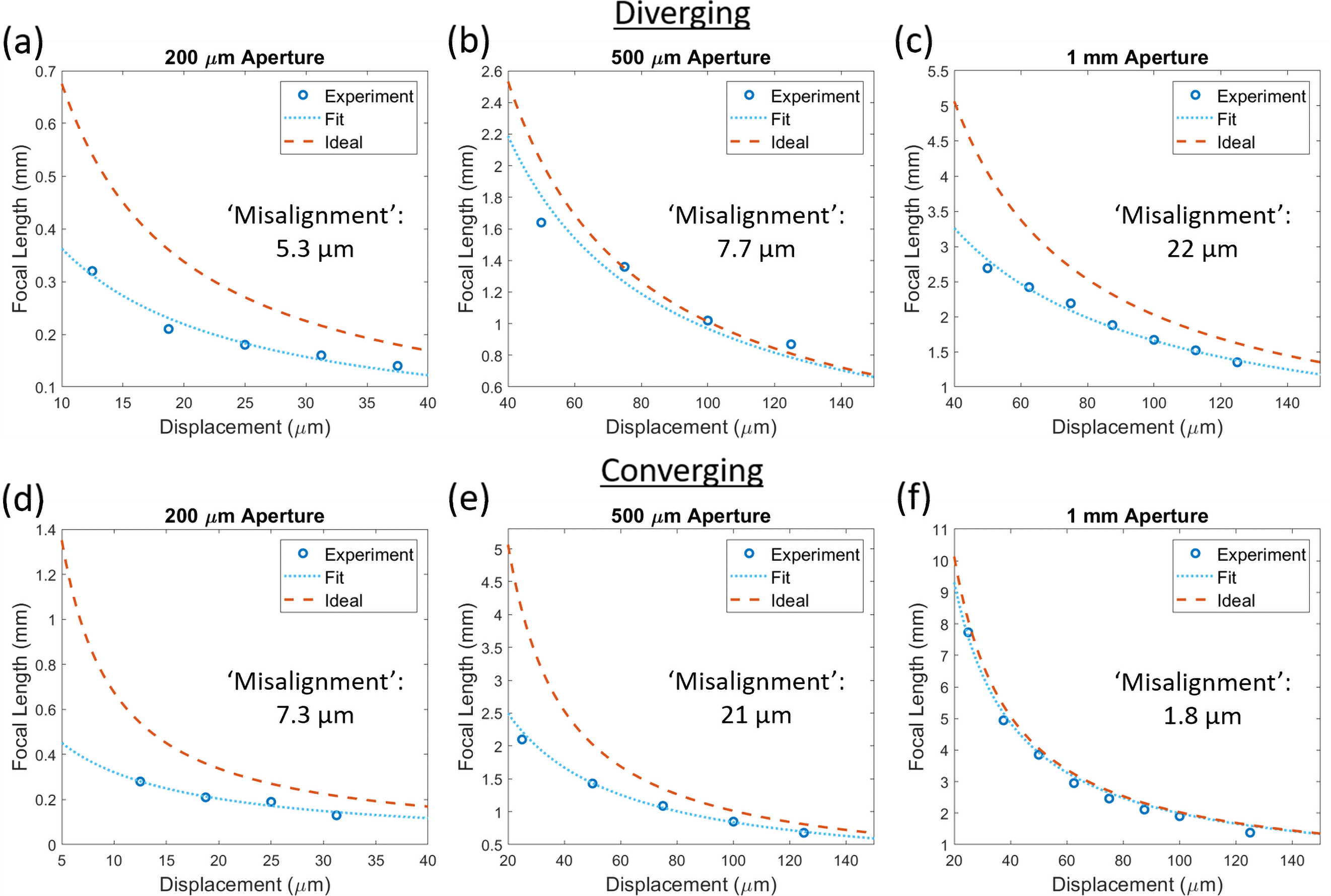


*Figure* ***S5.*** *Focal tuning by manually displacing the Alvarez metasurfaces relative to each other, on the diverging side with an aperture size of (a) 200 µm, (b) 500 µm, and (c) 1 mm, and on the converging side with an aperture size of (d) 200 µm, (e) 500 µm, and (f) 1 mm, compared to the corresponding ideal Alvarez behaviors. The ‘misalignment’ values estimate the deviation of the fitted Alverez metalens tuning behavior from the ideal designed Alvarez tuning behavior.*

We measured the performance of Alvarez metasurface lenses via manual tuning to investigate the causes of the observed deviation in measured focal tuning behavior of the MEMS Alvarez metalens from the designed Alvarez behavior under ideal conditions. These devices consisted of Alvarez metalenses fabricated with the same metasurface designs for the 200 µm aperture (as reported in the main text) and additional Alvarez metalens with increased aperture sizes of 500 µm and 1 mm but without the MEMS actuator structures. Two complementary Alvarez metasurfaces were mounted on two 3-axis translational stages as shown in the setup (Figure S3). The metasurfaces were then manually brought against each other with a negligible axial separation gap, aligned and then actuated laterally for equal displacement in opposite directions to generate focal tuning. Due to the accuracy limitation of manual actuation, larger ranges of displacement were introduced, leading to larger focal tuning ranges than the MEMS Alvarez metalens reported in the main text. In the manual experiments, the tuning behaviors on both the diverging and converging sides of the Alvarez lens were investigated, as shown in Figure S5, by introducing center-to-center offsets between the metasurfaces in the opposite directions.

Figure S5 shows the recorded shifts in focal position along the optical axis as a function of manually actuated lateral center-to-center displacements between the metasurfaces. In each set we plot the experimental data, a fitted curve accounting for lateral misalignment, and also the corresponding ideal behavior of the Alvarez lenses designed with a negligible separation gap, which is governed by the Alvarez lens equation^2^

$$f\left( d \right)= \frac{\pi}{2\lambda Ad}$$

where $f$ denotes the focal length, $\lambda$ the operating wavelength, $A$ the design cubic parameter, and $d$ the displacement translated by the complementary metasurfaces in the opposite directions giving a total center-to-center offset of $2d$.

The manually tuned focal lengths of the 200 µm metalens at small displacements are similar to the corresponding focal lengths electrostatically tuned in the MEMS-actuated Alvarez metalens with an initial center-to-center offset of 20 µm (details in the main text), showing that the MEMS integration process has limited effect on the tuning behavior deviation and therefore probably not the main cause. Meanwhile, all the manually tuned focal positions with various aperture sizes have exhibited smaller values than the corresponding ideal predictions.

While fitting the measured data to the Alvarez tuning curve, a ‘misalignment’ value $\Delta d$ was calculated as an estimation of the misalignment introduced during manual assembly of the Alvarez metalens, assuming only translational misalignment along the actuation direction, hence causing the deviation of the fitted experimental Alvarez curve from the ideal Alvarez curve following the relation

$$f\left( d \right)= \frac{\pi}{2\lambda A\left( d+\Delta d \right)}$$

The discrepancies in the ‘misalignment’ values for the same Alvarez lens (for diverging and the converging configuration, see Figure S5) indicate that there could be misalignment in directions other than translational. Nevertheless, these ‘misalignment’ values can act as crude quantitative estimates of the extent of total misalignment between the metasurfaces and can be used for preliminary behavior analysis across different aperture sizes.

During the design of Alvarez metasurfaces, we chose various cubic parameters (values of $A$) for each aperture size to achieve desirable tuning behaviors – typically a smaller cubic value for a larger aperture – which is also likely to affect the device tolerance on misalignment.

Meanwhile, Figure S5a and S5b show that an Alvarez metalens with a larger aperture generally has a higher tolerance to misalignment and can still perform much better (closer to the ideal scenario) than one with a smaller aperture and a smaller misalignment. However, the comparison between Figure S5b and S5c also shows that such increased tolerance due to larger aperture still has its limit, especially when the misalignment in the optical element is significantly worse. In conclusion, the quantitative comparison between the fit and ideal curves show that the deviation between the experiment and theory increases as the aperture size of metasurfaces decreases, probably with lower tolerance to misalignment overall.

## References:

1 Taylor, J. *Introduction to error analysis, the study of uncertainties in physical measurements*. 174-176 (1997).

2 Colburn, S., Zhan, A. & Majumdar, A. Varifocal zoom imaging with large area focal length adjustable metalenses. *Optica* **5**, 825-831, doi:10.1364/OPTICA.5.000825 (2018).

3 Yu, N. *et al.* Light propagation with phase discontinuities: generalized laws of reflection and refraction. *science* **334**, 333-337 (2011).
